# Retracted: Exogenous IL-4-Expressing Bone Marrow Mesenchymal Stem Cells for the Treatment of Autoimmune Sensorineural Hearing Loss in a Guinea Pig Model

**DOI:** 10.1155/2017/9539385

**Published:** 2017-07-13

**Authors:** BioMed Research International

BioMed Research International has retracted the article titled “Exogenous IL-4-Expressing Bone Marrow Mesenchymal Stem Cells for the Treatment of Autoimmune Sensorineural Hearing Loss in a Guinea Pig Model” [1]. Figures 3(a) and 3(b) in this article are identical to Figures 2(a) and 2(b) in the published article by Chang-qiang Tan, Xia Gao, Wen-jun Cai, Xiao-yun Qian, Ling Lu, and He Huang, “Experimental Study of Local Inner Ear Gene Therapy for Controlling Autoimmune Sensorineural Hearing Loss,” BioMed Research International, vol. 2014, Article ID 134658, 10 pages, 2014. doi: 10.1155/2014/134658, but they represent different experiments.
